# Differential Roles for Six P-Type Calcium ATPases in Sustaining Intracellular Ca^2+^ Homeostasis, Asexual Cycle and Environmental Fitness of *Beauveria bassiana*

**DOI:** 10.1038/s41598-017-01570-1

**Published:** 2017-05-03

**Authors:** Jie Wang, Xiao-Guan Zhu, Sheng-Hua Ying, Ming-Guang Feng

**Affiliations:** 1 Institute of Microbiology, College of Life Sciences, Zhejiang University, Hangzhou, Zhejiang 310058 China; 2 College of Food Science, South China Agricultural University, Guangzhou, 510642 Guangdong China

## Abstract

A global insight into the roles of multiple P-type calcium ATPase (CA) pumps in sustaining the life of a filamentous fungal pathogen is lacking. Here we elucidated the functions of five CA pumps (Eca1, Spf1 and PmcA/B/C) following previous characterization of Pmr1 in *Beauveria bassiana*, a fungal insect pathogen. The fungal CA pumps interacted at transcriptional level, at which singular deletions of five CA genes depressed *eca1* expression by 76–98% and deletion of *spf1* resulted in drastic upregulation of four CA genes by 36–50-fold. Intracellular Ca^2+^ concentration increased differentially in most deletion mutants exposed to the stresses of Ca^2+^, EDTA chelator, and/or endoplasmic reticulum and calcineurin inhibitors, accompanied with their changed sensitivities to not only the mentioned agents but also Fe^2+^, Cu^2+^ and Zn^2+^. Liquid culture acidification was delayed in the Δ*spf1*, Δ*pmr1* and Δ*pmcA* mutants, coinciding well with altered levels of their extracellular lactic and oxalic acids. Moreover, all deletion mutants showed differential defects in conidial germination, vegetative growth, conidiation capacity, antioxidant activity, cell wall integrity, conidial UV-B resistance and/or virulence. Our results provide the first global insight into differential roles for six CA pumps in sustaining intracellular Ca^2+^ level, asexual cycle and environmental fitness of *B*. *bassiana*.

## Introduction

P-type calcium ATPase (CA) pumps are a family of proteins that sustain intracellular Ca^2+^ homeostasis for normal functions of fungal cells and are classified to different groups in terms of subcellular localization to plasma membrane (PM), endoplasmic reticulum (ER) and Golgi apparatus (GA). Those localized in PM, smooth ER and GA secretory pathway (SP) are classified to the PMCA, SERCA and SPCA groups respectively^1–3^. *Saccharomyces cerevisiae* harbors Pmc1 (PMCA), Pmr1 (SPCA) and Spf1 (distinct from SPCA), but lacks SERCA, such as Eca1 or Nca-1 existing in filamentous fungi. The yeast Pmc1 may act as a vacuolar Ca^2+^ pump by transporting Ca^2+^ into vacuoles for the control of cytosolic Ca^2+^ level because *pmc1* deletion resulted in a large reduction in vacuolar Ca^2+^ pool and hence a poor growth under a Ca^2+^ stress to activate calcineurin, a Ca^2+^/calmodulin-activated Ser/Thr protein phosphatase, despite little effect on mating, sporulation and starvation under normal conditions^4^. Deletion of *spf1* caused disturbed Ca^2+^ homeostasis and upregulated expression of calcium-dependent response element (CDRE) genes while double deletion of *spf1* and *pmr1* resulted in increased calcium influx and hence elevated Ca^2+^ level in the yeast cells^5^. Deletion of *spf1* in *Candida albicans* resulted in defects in hyphal growth, development, biofilm formation and virulence as well as hypersensitivities to high levels of Ca^2+^ and ER stressors^6^.

Filamentous fungi have more PMCAs than yeast. For instance, *Aspergillus fumigatus* has three Pmc paralogues (PmcA–C), of which only PmcC is indispensable for the fungal life due to the lethality of its deletion while PmcA and PmcB function in sustaining intracellular Ca^2+^ and Mn^2+^ levels aside from a remarkable contribution of PmcA to the fungal virulence^7^. Two Pmc paralogues also exist in *Neurospora crassa* (Nca-2/3)^8^ but only Nca-2 was proven to function like the yeast Pmc1, contrasting to no phenotypic changes observed in the Δ*nca*-*3* mutant^9^. Fungal Eca1 homologues are highly conserved Ca^2+^ pumps crucial for the Ca^2+^ homeostasis between ER and cytosol due to a capability of their transporting Ca^2+^ from cytosol to ER^6, 10^. Deletion of *eca1* in *Cryptococcus neoformans* led to hypersensitivities to calcineurin inhibitors, ER inhibitors and osmotic agents as well as reduced thermotolerance and attenuated virulence^10^. Function loss of Eca1 in *Ustilago maydis* increased cytosolic Ca^2+^ levels, followed by severe defects in growth, morphology, and tolerance to high temperature and ER stress^11^, whereas the Eca1 homologue Nca-1 was shown to play no obvious role in *N*. *crassa* ^9^. A Pmr1 orthologue has been shown to be vital for vegetative growth, asexual development, multiple stress tolerance and virulence in *Beauveria bassiana* ^12^. These studies demonstrate that most CA pumps participate in a variety of cellular events and processes despite their functional variability in different fungi. In many filamentous fungal pathogens, however, not all CA pumps have been characterized for a global insight into their biological significance. Nor is it clear that multiple CA pumps are functionally dependent on or independent of one another in the fungal pathogens.

Filamentous fungal insect pathogens, such as *B*. *bassiana* that usually survive in asexual cycle *in vitro* or *in vivo*, are important biological control agents against arthropod pests, and their pest control potential depends on not only virulence but cell tolerance to environmental stresses, such as high temperature, solar UV irradiations and applied agrochemicals^13, 14^. There exist six genes encoding one SERCA (Eca1), three PMCAs (PmcA–C), one SPCA (Pmr1) and Spf1 orthologue in the genome database of *B*. *bassiana* ^15^. Of those, only Pmr1 has been functionally characterized^12^. In this study, we tried to elucidate the functions of five other CA pumps by multi-phenotypic analyses of single-gene deletion mutants with an emphasis being placed upon intracellular Ca^2+^ homeostasis, cellular responses to various metal ions and ER stressors. The previous Δ*pmr1* mutant was also included in this study for comparison. Our results provide a global insight into the vital, but differential, roles for all CA pumps in sustaining not only intracellular Ca^2+^ homeostasis but also asexual cycle, antioxidant activity, cell wall integrity and pest control potential of *B*. *bassiana*.

## Results

### Bioinformatic features of six CA pumps in *B*. *bassiana*

Six CA pumps were located in the *B*. *bassiana* database^15^ through online blast analysis using the queries of all CA sequences in *A*. *fumigatus*, *C*. *albicans* and *S*. *cerevisiae*. Three of the located pumps are Eca1, Pmr1 and Spf1 orthologues (NCBI accession codes: EJP63025, EJP63084 and EJP65528) and consist of 998, 2339 and 1318 amino acids with molecular masses of 107.88, 253.4 and 146.79 kDa, respectively. Other three CA pumps (NCBI accession codes: EJP70918, EJP64689 and EJP63186) are homologous to PmcA, PmcB and PmcC in *A*. *fumigatus* and composed of 1269, 1379 and 1155 amino acids with molecular masses of 140.94, 149.98 and 124.53 kDa, respectively. As illustrated in Fig. S1, all of the located CA pumps possess an E1–E2 ATPase domain and a haloacid dehalogenase-like hydrolase domain (HAD), which are typical for the CA family. The Eca1 orthologue harbors uniquely an N-terminal ER-targeting pentapeptide motif typical for the SERCA group^16^ and a C-terminal motif (KKNL) in association with canonical dilysine ER retention^17^. Three Pmc paralogues are structurally similar to each other with an exception of PmcB lacking an N-terminal cation transporter ATPase domain and share 41–60% sequence identity with the counterparts in *A*. *fumigatus*. Besides these conserved domains, the Pmr1 orthologue has two additional motifs (COG4 and SUAS) at C-terminus. The Spf1 orthologue is structurally typical for the subfamily of P-type-V ATPases with a single catalytic subunit^18^ and shows a sequence identity of 53% to the *S*. *cerevisiae* Spf1.

### Transcriptional interaction of CA pumps

Each CA gene was deleted from the wild-type strain *B*. *bassiana* ARSEF 2860 (designated WT herein) by homologous recombination of its 5′ and 3′ coding/flanking fragments separated by the *bar* marker and rescued by integrating ectopically the cassette of its full-length sequences and the *sur* marker into the deletion mutant. The expected recombination events were confirmed via PCR and Southern blotting analyses (Fig. S2A–F).

**Figure 1:**
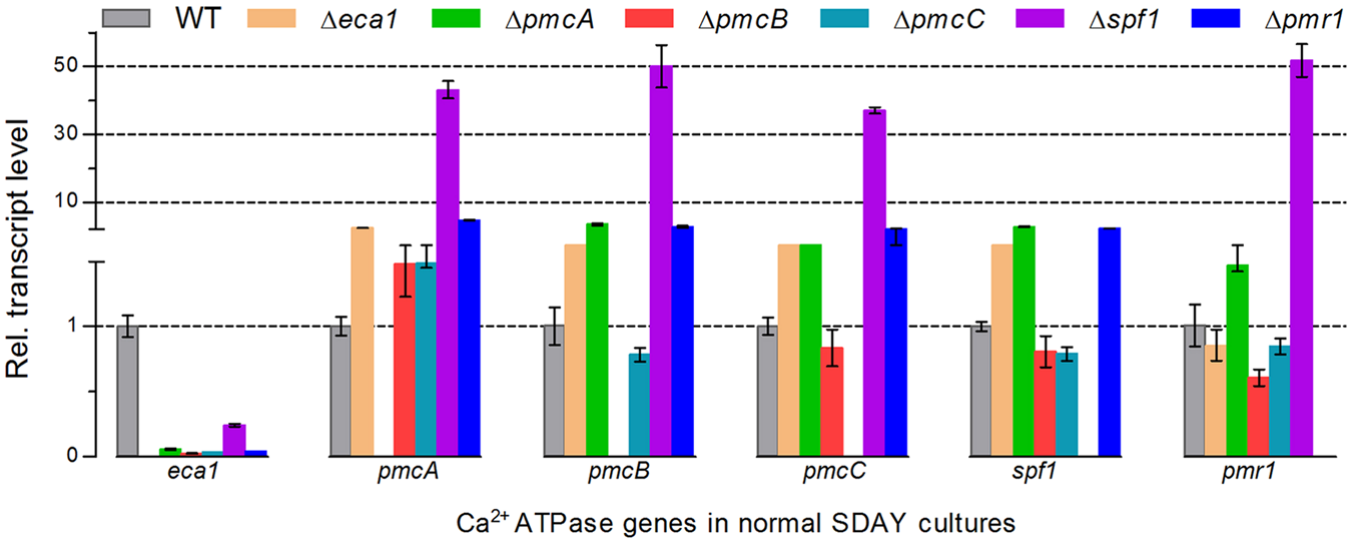
Relative transcript levels of CA genes in the 3-day-old SDAY cultures of their deletion mutants versus the wild-type *B*. *bassiana* strain. Note that the transcript of each CA gene was undetectable in its deletion mutant and that the deletion of each gene resulted in drastic transcript changes of some others. Error bars: SD of the mean from three cDNA samples of each strain detected in qRT-PCR experiments with paired primers (Table S1).

As a consequence, the transcript of each deleted CA gene was undetectable in quantitative real-time PCR (qRT-PCR) experiments, indicating again a success for each deletion. Intriguingly, expression of some CA genes was drastically altered in the 3-day-old cultures of each deletion mutant with respect to the WT standard. As shown in Fig. 1, transcript level of *eca1* decreased by 76% in Δ*spf1* and 94–98% in the deletion mutants of four other CA genes. Deletion of *spf1* increased transcript levels of *pmcA*–*C* and *pmr1* by 36- to 50-fold. The latter CA genes were also differentially upregulated by 0.5–3.7-fold in the absence of *eca1*, *pmcA* or *pmr1*. In both Δ*pmcB* and Δ*pmcC*, only *pmcA* was upregulated by 48% while other CA genes except *spf1* were slightly downregulated. These changes implied that some members in the CA family of *B*. *bassiana* could interact at transcriptional level and that Eca1 was most sensitive to the absence of each other member. The absence of *spf1* was compensated by the drastic upregulation of *pmr1* and three *pmc* genes, which were not or weakly compensatory for the absence of one another.

### The CA pumps sustaining intracellular Ca^2+^ homeostasis and cell tolerance to metal ions and ER/calcineurin inhibitors

**Figure 2:**
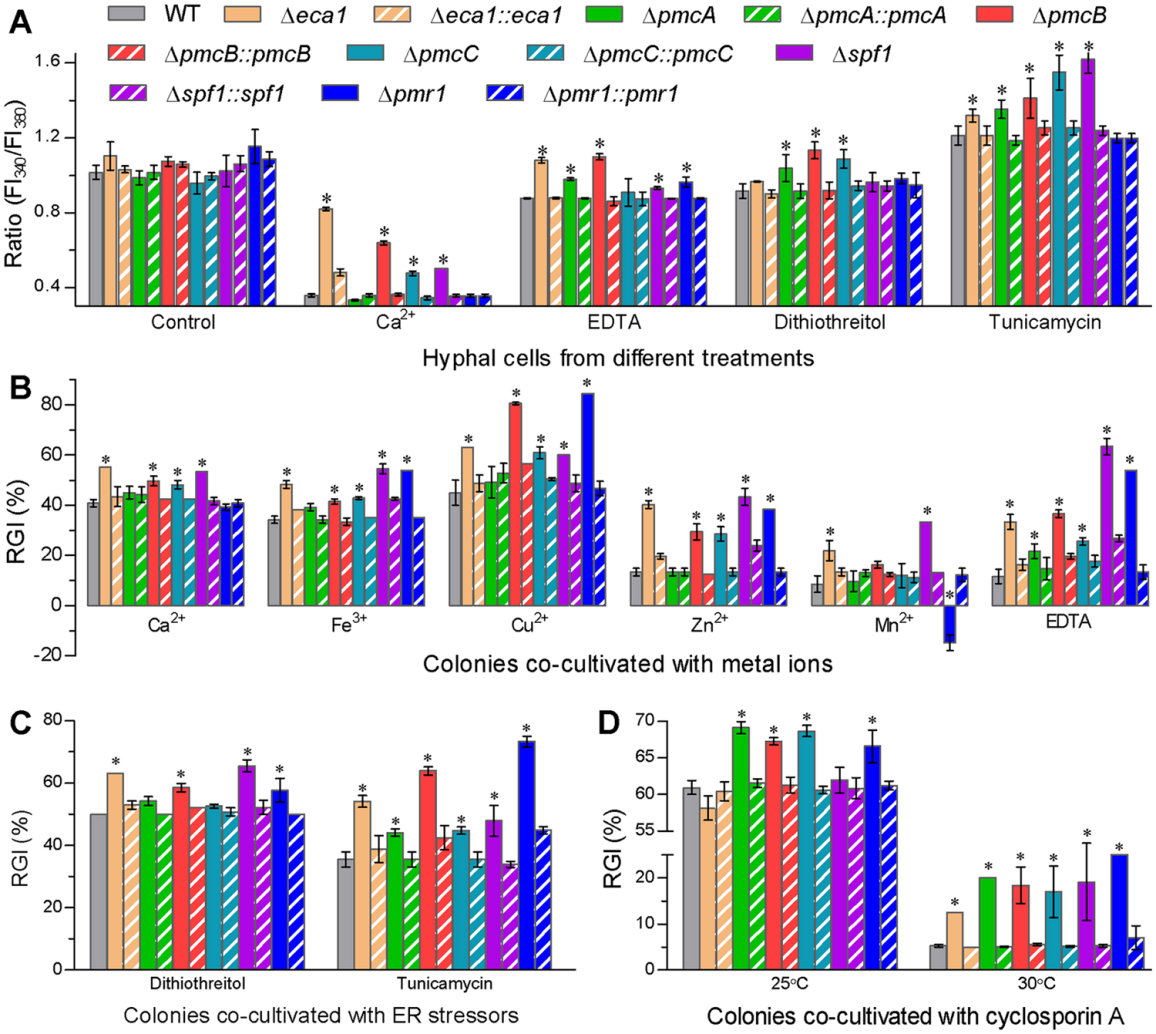
Roles for six CA pumps in sustaining intracellular Ca^2+^ homeostasis and cellular responses to metal ions, and ER and calcineurin inhibitors in *B*. *bassiana*. (**A**) FI_340_/FI_380_ ratios indicative of free Ca^2+^ concentrations in the cells of CA deletion mutants and control strains stained with Fura-2-AM. (**B**–**D**) Relation growth inhibition of fungal colonies by metal ions [Ca^2+^ (0.5 M), Fe^2+^ (3 mM), Cu^2+^ (2 mM), Zn^2+^ (10 mM), Mn^2+^ (5 mM) and the metal ion chelator EDTA (2 mM)], ER inhibitors [dithiothreitol (10 mM) and tunicamycin (5 µg/ml)] and calcineurin inhibitor [cyclosporin A (25 ng/ml)] in minimal CZA, respectively. All colonies initiated with the spotting method were incubated for 8 days at 25 °C. The asterisked bars in each bar group differ significantly from those unmarked (Tukey’s HSD, *P* < 0.05). Error bars: SD from three cell samples (**A**) or replicates (**B**–**D**).

The deletion mutants showed differential disturbance in intracellular Ca^2+^ homeostasis. The free Ca^2+^ concentration was quantified as a ratio of fluorescence intensities from the cells stained with Fura-2 acetoxymethyl ester (Fura-2-AM) and assessed at two excitation wavelengths^7^. The ratio was significantly higher in the cells of most CA mutants than of the WT after 30 min exposure to different treatments (Tukey’s HSD, *P* < 0.05) but was of little variability among all the tested strains under control conditions (Fig. 2A). The Ca^2+^ level increased by 129%, 78%, 33% and 40% in the respective cells of Δ*eca1*, Δ*pmcB*, Δ*pmcC* and Δ*spf1* treated with Ca^2+^ (0.5 M), 6–25% in five Δ *pmcC*-exclusive CA mutants treated with ETDA (2 mM), 14–24% in the Δ*pmcA*–*C* mutants treated with dithiothreitol (10 mM), and 9–34% in five Δ*pmr1*-exclusive CA mutants treated with tunicamycin (5 µg/ml). These increased ratios indicated differential facilitation of exogenous Ca^2+^ accumulation in the hyphal cells of the CA mutants exposed to different treatments.

The deletion mutants exhibited significant changes (Tukey’s HSD, *P* < 0.05) in sensitivities to metal ions and EDTA during growth on minimal Czapek-Dox agar (CZA) (Fig. 2B). Inclusion of Ca^2+^ in CZA suppressed the growth of Δ*eca1*, Δ*pmcB*, Δ*pmcC* and Δ*spf1* by 7–14% as compared with the WT growth. The four mutants and Δ*pmr1* were also 7–20%, 15–40%, 15–30% and 13–51% more sensitive to Fe^2+^, Cu^2+^, Zn^2+^ and EDTA, respectively. Mn^2+^ added to the medium reduced the growth of Δ*eca1* by 13% and of Δ*spf1* by 25% but uniquely facilitated the Δ*pmr1* growth by 23%. The Δ*pmcA* mutant showed an increased (10%) sensitivity to only EDTA. For all tested strains, interestingly, intracellular Ca^2+^ concentrations were linearly correlated with cellular sensitivities to the Ca^2+^ stress during colony growth (r^2^ = 0.71, *F*_1,11_ = 26.9, *P* = 0.0003). This suggests a significant link of the disturbed Ca^2+^ homeostasis with their sensitivities to elevated external Ca^2+^ level.

We also assessed the responses of each strain to two ER inhibitors and cyclosporin A (CsA), an immunosuppressive drug that inhibits calcineurin signaling by forming a complex with the immunophilin cyclophilin^19^. As a result, the Δ*pmcA*, Δ*pmcB*, Δ*pmcC* and Δ*pmr1* mutants exhibited a significant, but mild, increase (6–13%) in sensitivity to either dithiothreitol (Fig. 2C) or CsA at 25 °C (Fig. 2D). The six deletion mutants were all more sensitive to tunicamycin (8–37%) at 25 °C and CsA at 30 °C (7–19%) than the WT. All of these phenotypic changes were well or largely restored by targeted gene complementation.

### The CA pumps acting in extracellular pH balance

**Figure 3:**
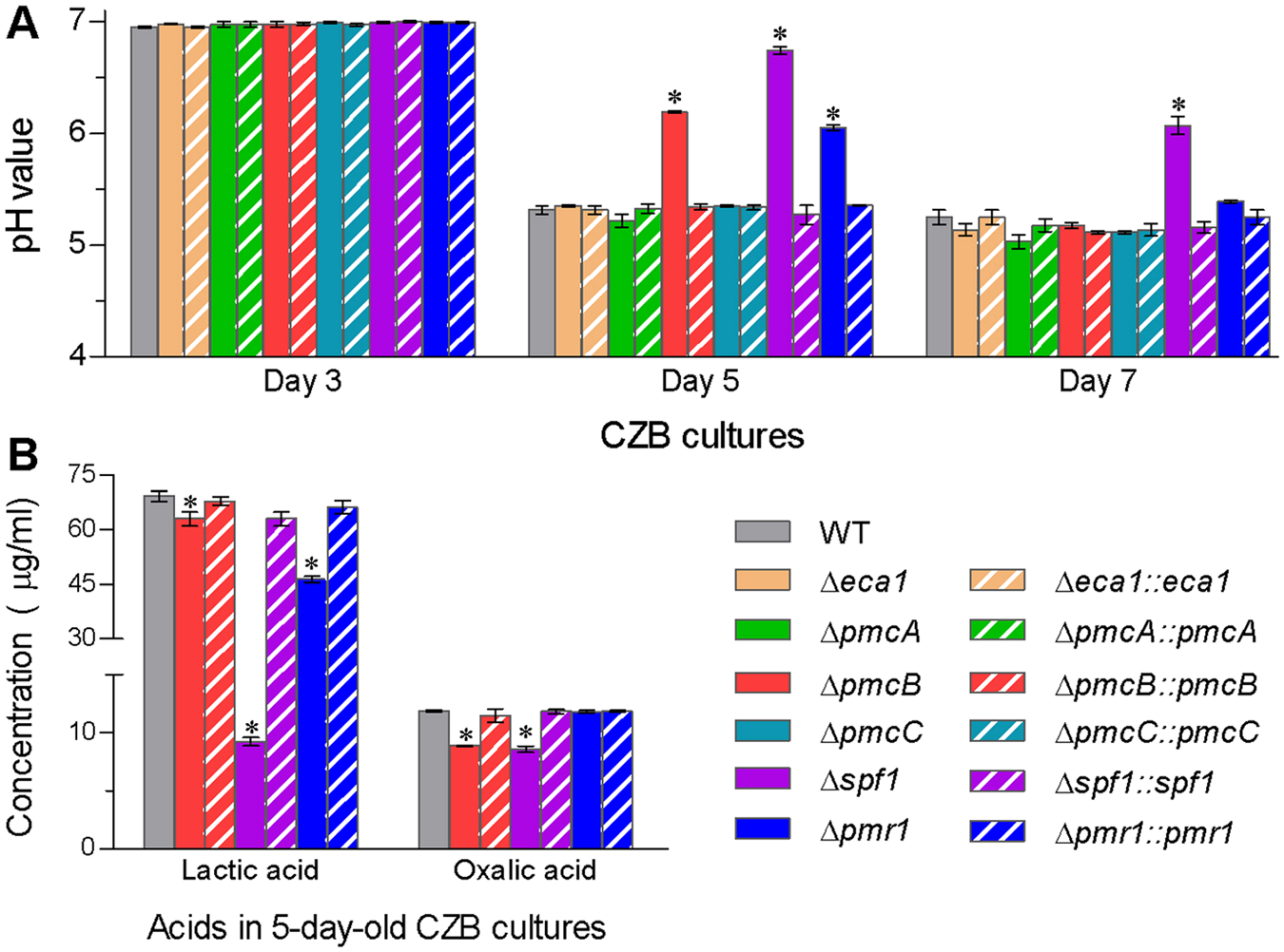
The CA pumps sustaining extracellular pH levels in *B*. *bassiana*. (**A**,**B**) pH levels measured from the supernatants of liquid cultures of different strains during incubation in minimal CZB (initial pH = 7.3) and concentrations of lactic acid and oxalic acid quantified from the supernatants of 5-day-old CZB cultures, respectively. The asterisked bars in each bar group differ significantly from those unmarked (Tukey’s HSD, *P* < 0.05). Error bars: SD from three replicates.

Incubation of conidial suspensions in CZB (agar-free CZA) resulted in a significant delay in the acidification of Δ*pmcA*, Δ*spf1* and Δ*pmr1* cultures (Fig. 3A) although extracellular pH levels did not differ between other deletion mutants and their control strains (WT and complementary strains). The three deletion mutants showed much higher pH levels in 5-day-old CZB cultures than their control strains. The pH level remained highest in the 7-day-old Δ*spf1* cultures but the pH difference between Δ*pmcA* or Δ*pmr1* and control strains disappeared on day 7. Quantification of organic acids in the 5-day-old cultures demonstrated that the concentrations of lactic acid and oxalic acid (Fig. 3B) decreased by 87% and 28% in Δ*spf1* and 9% and 25% in Δ*pmcA*, respectively, as compared with the WT. In Δ*pmr1*, only lactic acid level was lowered by 32%. Concentrations of other organic acids, including malic acid, pyruvix acid and citric acid, did not differ significantly between any deletion mutant and control strains (data not shown). Apparently, the most delayed acidification in the Δ*spf1* culture was attributable to much less accumulation of extracellular organic acids, particularly lactic acid. Overall, Spf1 made the most prominent contribution to extracellular pH balance in *B*. *bassiana*, followed by Pmr1 and PmcA.

### The CA pumps sustaining asexual cycle *in vitro*

**Figure 4:**
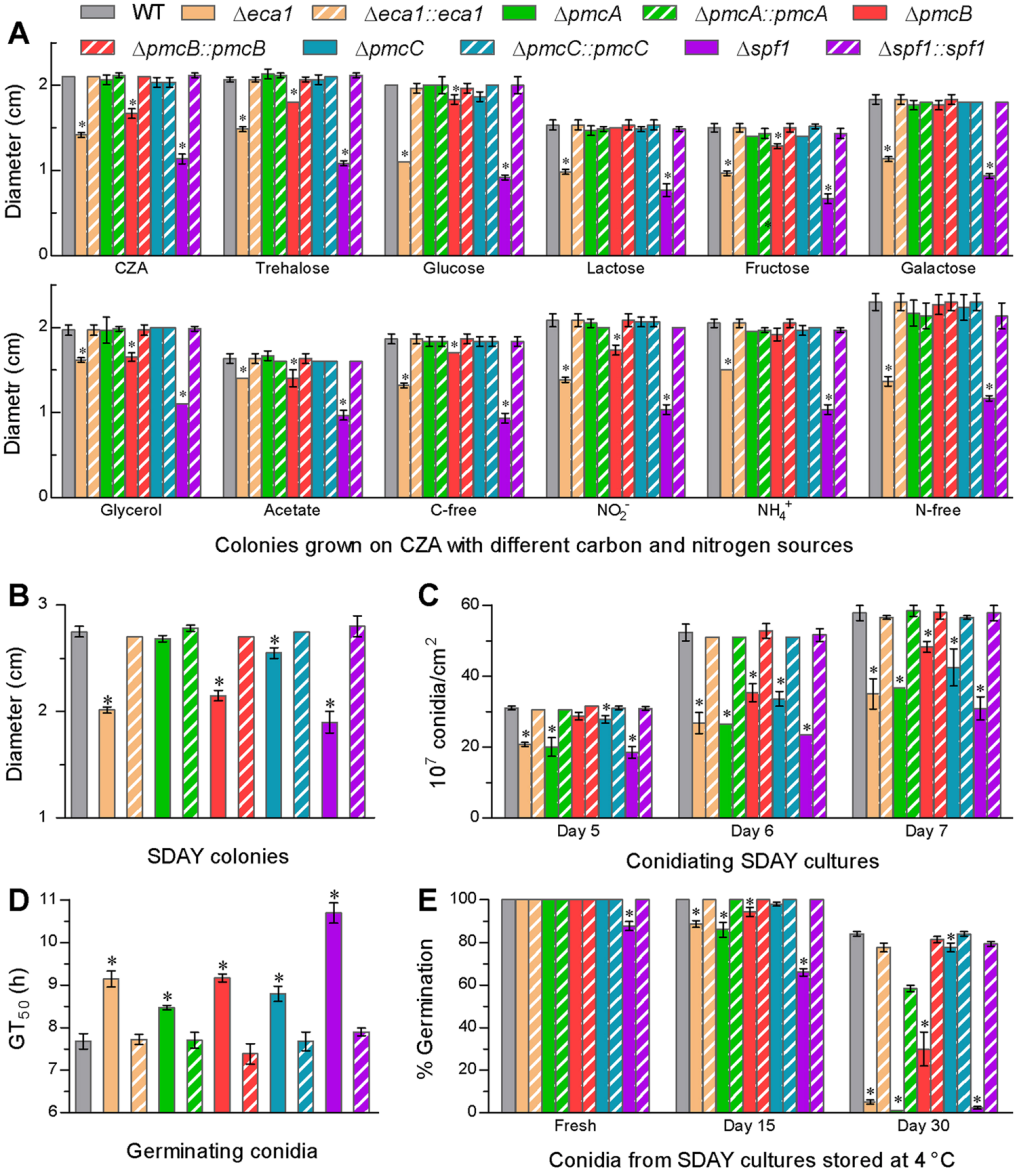
The CA pumps sustaining vegetative growth, conidiation capacity and conidial viability in *B*. *bassiana*. (**A**,**B**) Diameters of fungal colonies after 8 days of cultivation at 25 °C on the plates of minimal CZA, modified CZA media with different carbon and nitrogen sources and rich SDAY, respectively. Each colony was initiated with the spotting method. (**C**) Conidial yields quantified over the days of normal cultivation on SDAY plates spread with 100 µl aliquots of conidial suspension for culture initiation. (**D**) Median germination time (GT_50_) estimated as an index of conidial germination rate for each of the fungal strains. (**E**) Germination percentages of conidia taken from fresh 7-day-old SDAY cultures and the same cultures stored at 4 °C for 15 and 30 days, after 24 h incubation at 25 °C. The asterisked bars in each bar group differ significantly from those unmarked (Tukey’s HSD, *P* < 0.05). Error bars: SD from three replicates.

Host infection by *B*. *bassiana* requires the fungal ability to make use of scant nutrients on the host integument where conidia adhere for germination and hyphal extension for cuticular penetration. For this reason, we assessed the growth rates of each strain on minimal CZA media with different carbon/nitrogen sources and availability. Compared to the WT, the Δ*eca1* and Δ*spf1* mutants displayed severe growth defects because their colonies initiated with 1 µl aliquots of conidial suspension diminished by 14–45% and 31–55%, respectively, on all minimal media after 8 days of normal incubation (Fig. 4A). The Δ*pmcB* colonies diminished mostly by 20% on the standard CZA and only 8–16% on some of the tested carbon and nitrogen sources. In contrast, no significant growth defect was observed in either Δ*pmcA* or Δ*pmcC* mutant on any minimal medium. Previously, the Δ*pmr1* colonies grown on the minimal media were 37–73% smaller than the WT counterparts^12^. The present and previous studies indicated that the fungal growth defect on a given minimal medium was most severe in Δ*pmr1*, followed by Δ*spf1*, Δ*eca1* and Δ*pmcB* in order. Similarly, the colonies of Δ*spf1*, Δ*eca1* and Δ*pmcB* grown on Sabouraud dextrose agar plus yeast extract (SDAY; standard medium for cultivation of fungal entomopathogens) were 31%, 27% and 22% smaller than the WT colonies (Fig. 4B), and their growth defects were also less severe than those observed in the Δ*pmr1* mutant (48%). Exceptionally, the growth defect of Δ*pmcC* on the rich medium was minor.

Conidial yields quantified from the SDAY cultures decreased differentially in Δ*eca1*, Δ*pmcA*, Δ*pmcB*, Δ*pmcC* and Δ*spf1* over the days of normal cultivation in comparison with the WT yield. The final yield reduction reached 46% in Δ*spf1*, 39% in Δ*eca1*, 36% in Δ*pmcA*, 26% in Δ*pmcC* and 16% in Δ*pmcB* (Fig. 4C). All these reductions were much lower than a 72% yield decrease in Δ*pmr1* ^12^. In addition, fresh conidia produced by the deletion mutants required significantly (0.7–3 h) longer time for 50% germination at 25 °C than those from the WT culture (Fig. 4D) although germination percentages within 24 h were not affected in all CA mutants except Δ*spf1*. However, most of the deletion mutants lost conidial viability much more rapidly than the control strains after their cultures were preserved for 15 or 30 days at 4 °C (Fig. 4E). The viability loss was most rapid in Δ*spf1*, relatively slow in Δ*pmcB* and slight in Δ*pmcC*.

All together, the *in vitro* asexual cycle of *B*. *bassiana* was severely affected in Δ*pmr1* and Δ*spf1*, followed by Δ*eca1* and Δ*pmcB*, but was less affected in Δ*pmcA* and Δ*pmcC*. The latter two mutants displayed only minor defects in some, but not all, of the examined phenotypes relevant to the fungal asexual cycle.

### The CA pumps sustaining antioxidant activity and cell wall integrity

**Figure 5:**
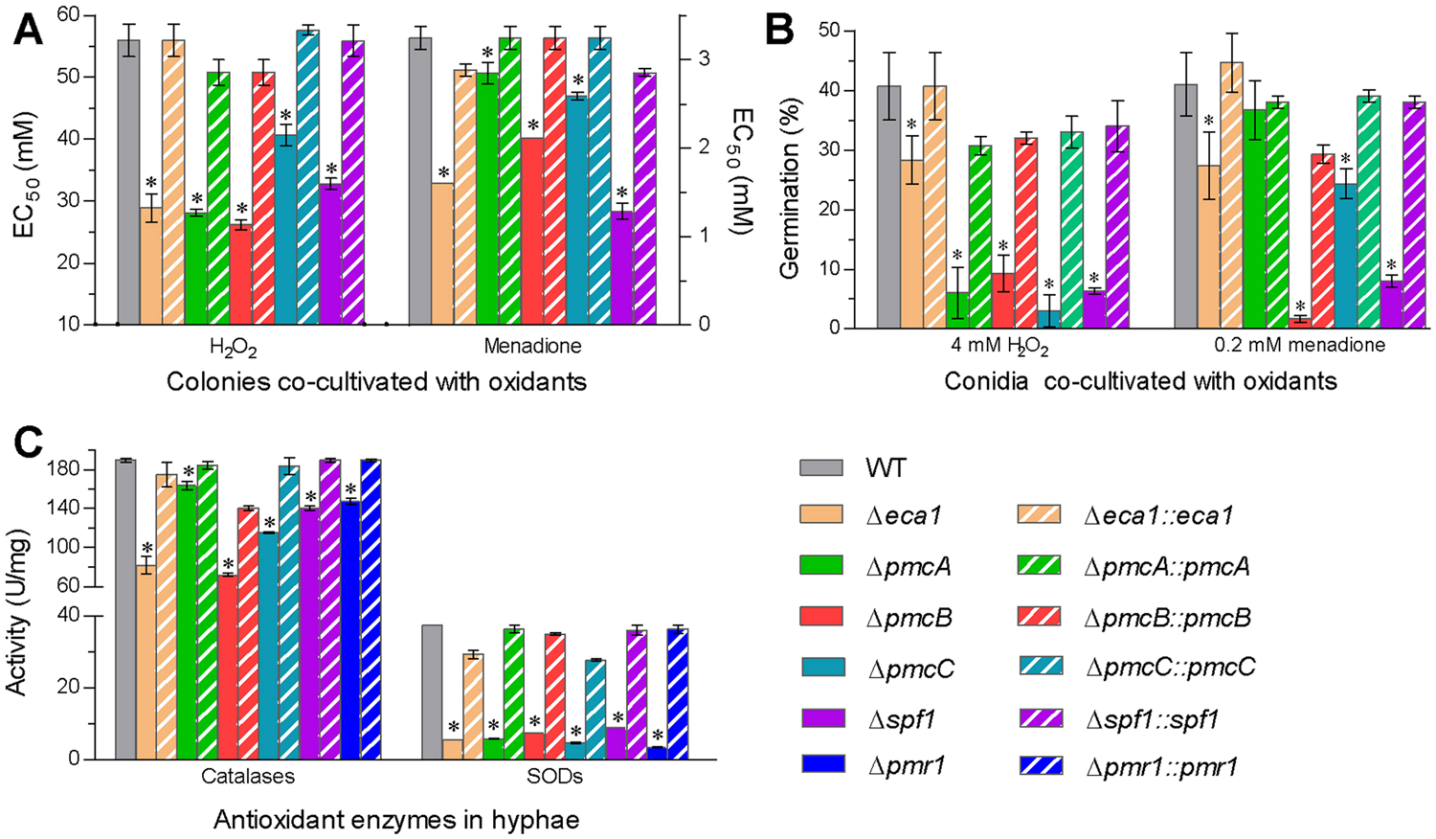
The CA pumps sustaining antioxidant activity in *B*. *bassiana*. (**A**) EC_50_s for H_2_O_2_ and menadione to suppress 50% growth of fungal colonies after 6 days of incubation at 25 °C on 1/4 SDAY plates, on which hyphal mass discs (5 mm diameter) were attached for colony initiation. (**B**) Germination percentages of fresh conidia after 24 h co-cultivation with H_2_O_2_ and menadione on a germination medium at 25 °C. (**C**) Total activities of SODs and catalases quantified from the protein extracts of 3-day-old SDAY cultures (hyphal cells). The asterisked bars in each bar group differ significantly from those unmarked (Tukey’s HSD, *P* < 0.05). Error bars: SD from three replicates.

As an index of antioxidant response, mean (±SD) EC_50_s required for H_2_O_2_ and menadione to suppress 50% growth of the WT on 1/4 SDAY (amended with 1/4 of each SDAY nutrient) at 25 °C were 55.9 ± 2.7 and 3.2 ± 0.1 mM (Fig. 5A), respectively. Compared to the WT estimates, all deletion mutants were significantly more sensitive to the two oxidants (Tukey’s HSD, *P* < 0.05). The EC_50_ estimates for H_2_O_2_ and menadione were lowered by 41% and 60% in Δ*spf1*, 48% and 51% in Δ*eca1*, 53% and 35% in Δ*pmcB*, 50% and 12% in Δ*pmcB*, and 27% and 20% in Δ*pmcC*, respectively. Co-cultivation of conidia with H_2_O_2_ (4 mM) and menadione (0.2 mM) also reduced the germination of all deletion mutants significantly more than that of the control strains (Fig. 5B). Conidial sensitivity to H_2_O_2_ increased similarly by 31–38% in four deletion mutants and 12% in Δ*eca1*. Conidial sensitivity to menadione increased by 33% and 39% in Δ*spf1* and Δ*pmcB*, and 14% and 17% in Δ*eca1* and Δ*pmcC*, respectively, but was not significantly affected in Δ*pmcA*. Previously, the sensitivities of Δ*pmr1* to H_2_O_2_ and menadione increased respectively by 34% and 46% during colony growth and 13% and 77% during conidial germination under the same culture conditions^12^. Moreover, total activities of catalases and superoxide dismutases (SODs) quantified in the crude extracts from 3-day-old WT cultures were averaged as 189.3 and 37.5 U/mg (Fig. 5C), respectively. Compared to these estimates, the SOD activity was reduced by 91% in Δ*pmr1*, 76% in Δ*spf1* and 81–87% in the rest CA mutants. The catalase activity decreased mostly in Δ*pmcB* (62%), followed by Δ*eca1* (57%), Δ*pmcC* (39%), Δ*spf1* (26%), Δ*pmr1* (22%) and Δ*pmcA* (14%) in order. For all the tested strains, intriguingly, a significantly linear correlation was found between their SOD activities and the EC_50_s of H_2_O_2_ (r^2^ = 0.61, *F*_1,11_ = 14.2, *P* = 0.0031) or menadione (*r* ^2^ = 0.47, *F*_1,9_ = 8.0, *P* = 0.0164) or between their catalase activities and the EC_50_s of H_2_O_2_ (*r* ^2^ = 0.81, *F*_1,11_ = 38.0, *P* = 0.0001) or menadione (r^2^ = 0.54, *F*_1,11_ = 10.7, *P* = 0.0075).

**Figure 6:**
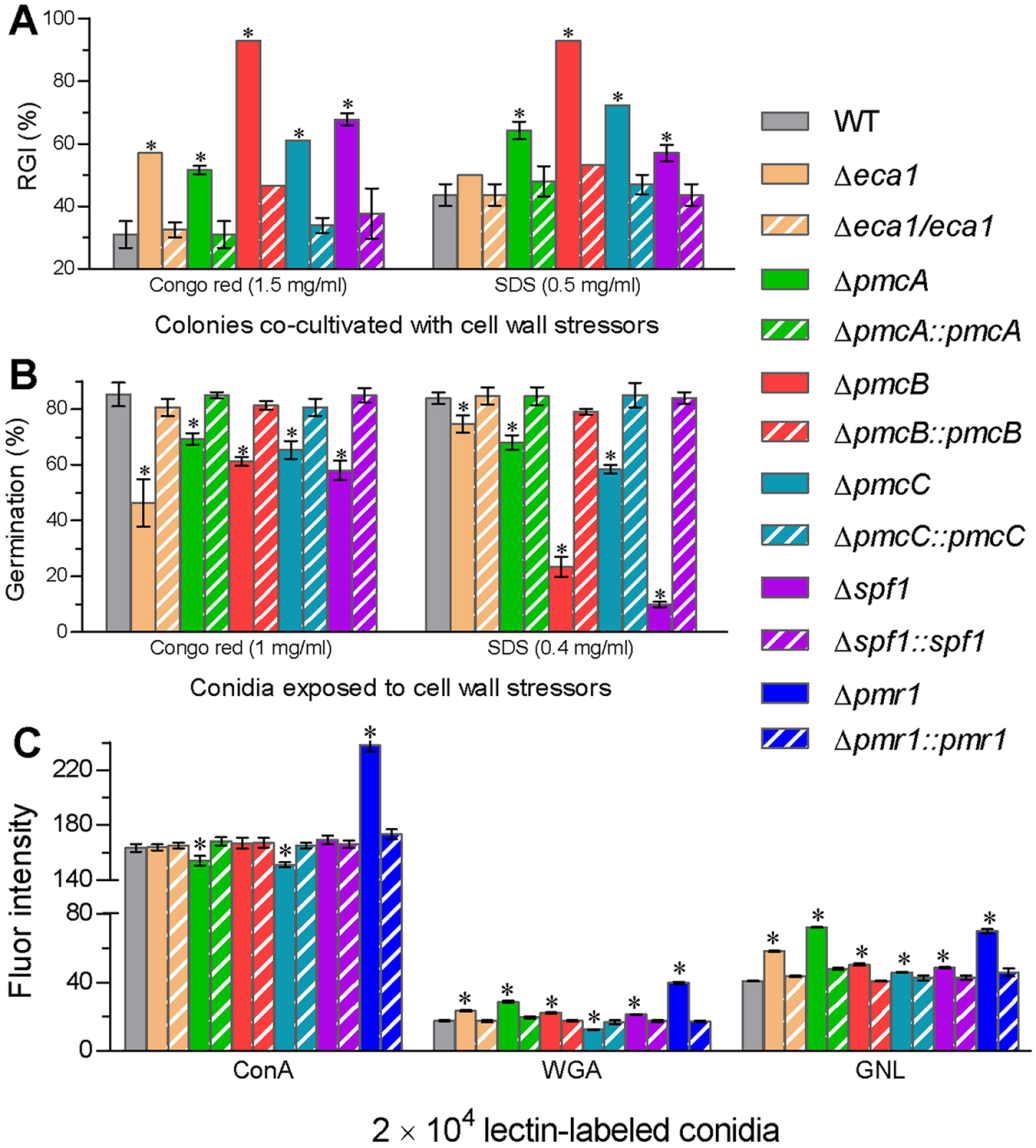
The CA pumps sustaining cell wall integrity in *B*. *bassiana*. (**A**,**B**) Relative inhibition of Congo red and SDS to fungal colony growth and conidial germination, respectively, at 25 °C on a medium containing a sensitive concentration of each cell wall perturbing chemical as indicated. (**C**) Fluorescence readings for the changes of cell wall components in the ConA-, WGA- and GNL-labeled conidia analyzed via flow cytometry. The asterisked bars in each bar group differ significantly from those unmarked (Tukey’s HSD, *P* < 0.05). Error bars: SD from three replicates.

Aside from the altered antioxidant responses, the deletion mutants were more sensitive to cell wall perturbation by Congo red and SDS than their control strains during colony growth and conidial germination. Relative inhibition of the WT growth by Congo red (1.5 mg/ml) and SDS (0.5 mg/ml) in 1/4 SDAY reached 31% and 44% (Fig. 6A), respectively. The inhibitory effect of Congo red on colony growth increased to 93% in Δ*pmcB* and 52–68% in the rest deletion mutants. Inclusion of SDS in the medium also suppressed 49% more the growth of Δ*pmcB* and 13–28% more the growth of Δ*pmcA*, Δ*pmcC* and Δ*spf1* than the WT growth despite little effect on the growth of Δ*eca1*. In addition, adding Congo red (1 mg/ml) and SDS (0.4 mg/ml) to a germination medium resulted in significantly (15–39% and 9–74%) lower conidial germination of the deletion mutants than of the control strains (Fig. 6B). The Δ*eca1* conidia were most sensitive to Congo red whereas conidial sensitivity to SDS increased drastically by 74% in Δ*spf1* and 61% in Δ*pmcB*. Previously, the Δ*pmr1* sensitivities to Congo red and SDS increased respectively by 55% and 12% during colony growth and 29% and 50% during conidial germination^12^.

The increased cellular sensitivity of each deletion mutant to cell wall perturbation implicated a possible alternation in cell wall composition. This speculation was confirmed in the assays of three fluorescence-labeled lectins bound to conidial surfaces. As illustrated in Fig. 6C, the cell wall components α-glucose and α- *N*-acetylglucosamine (α-GlcNAc) in the conidia labeled by the lectin concanavalin A (ConA) increased by 45% in Δ*pmr1* and decreased by 6% and 8% in Δ*pmcA* and Δ*pmcC* versus the WT. The components β-GlcNAc and sialic acids labeled by wheat germ agglutinin (WAG) increased mostly by 124% in Δ*pmr1*, followed by 62% in Δ*pmcA* and 19–32% in three other mutants, but decreased by 30% in Δ*pmcC*. The components mannose residues labeled by *Galanthus nivalis* lectin (GNL) increased by 77% in Δ*pmcA*, 71% in Δ*pmr1*, 42% in Δ*eca1*, and 12–23% in the rest deletion mutants. All of these cell wall changes were well restored in complementary mutants and hence indicated vital, but differential, roles for the CA pumps in sustaining the cell wall integrity of *B*. *bassiana*.

### The CA pumps sustaining pest control potential of *B*. *bassiana*

**Figure 7:**
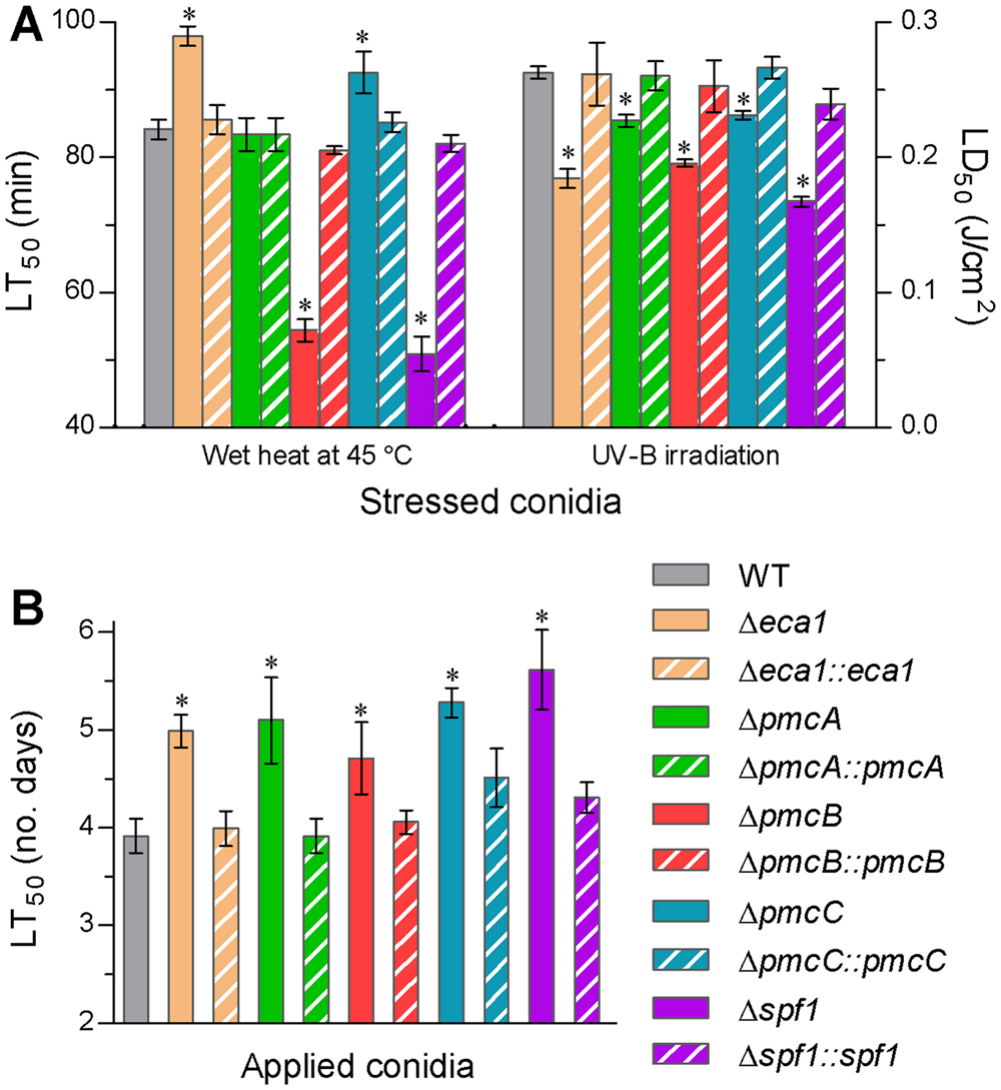
The CA pumps sustaining biological control potential in *B*. *bassiana*. (**A**,**B**) LT_50_ (min) and LD_50_ (J/cm^2^) estimated for conidial tolerance of each strain to 45 °C wet-heat stress and UV-B irradiation, respectively. (**C**) LT_50_ (no. days) estimated for the virulence of each strain to *G*. *mellonella* larvae infected by topical application of a 10^7^ conidia/ml suspension. The asterisked bars in each bar group differ significantly from those unmarked (Tukey’s HSD, *P* < 0.05). Error bars: SD from three replicates.

Aerial conidia produced on solid substrates are active ingredients of *B*. *bassiana* formulations as fungal insecticides^20^ but are inevitably exposed to outdoor stresses of high temperature and solar UV-B irradiation upon field application. Previously, LT_50_ for conidial tolerance to wet-heat stress at 45 °C and LD_50_ for conidial resistance to UV-B irradiation were reduced respectively by 41% and 51% in the Δ*pmr1* mutant versus the WT^12^. In this study, LT_50_ and LD_50_ for thermotolerance and UV-B resistance of the WT conidia were assessed as 84 min and 0.26 J/cm^2^ (Fig. 7A), respectively. Compared to the WT estimates, conidial tolerance to the wet-heat stress decreased by 40% in Δ*spf1* and 35% in Δ*pmcB* but increased significantly by 16% in Δ*eca1* and 10% in Δ*pmcC* and was unaffected in Δ*pmcA*. Conidial UV-B resistance was mostly lowered by 36% in Δ*spf1*, followed by 30% in Δ*eca1*, 26% in Δ*pmcB*, and 12–14% in Δ*pmcA* and Δ*pmcC*. In standardized bioassays, median lethal time (LT_50_) for the WT strain to kill 50% of *Galleria mellonella* larvae was averaged as 3.9 ± 0.2 days (Fig. 7B). Compared to this estimate, LT_50_ was significantly (20–43%) longer in all deletion mutants except Δ*pmr1*, which had an LT_50_ as long as twice of the WT estimate against the same insect species^12^.

## Discussion

As shown above, all CA pumps play important roles in sustaining cellular events and life cycle of *B*. *bassiana*, and most of them may interact with one another at transcriptional level. The expression of Eca1 is dependent on the functional normality of five other CA pumps because its transcript level was drastically reduced by the deletion of each other CA gene. Function loss of Spf1, Pmr1, Eca1 or PmcA can be differentially compensated by upregulated expression of four CA genes excluding *eca1*. Such transcriptional interaction has been rarely shown in previous studies. Perhaps for this reason, the CA-coding genes are all dispensable for the life of *B*. *bassiana* due to no lethality attributed to any targeted gene deletion. This is different from a lethality attributed to the deletion of *pmcC* in *A*. *nidulans* ^7^. The *B*. *bassiana* CA pumps examined in this study were important for intracellular Ca^2+^ homeostasis in response to Ca^2+^, EDTA and/or two ER inhibitors. Exceptionally, intracellular Ca^2+^ level increased in the response of Δ*pmr1* to only EDTA, but was not affected in the response of Δ*eca1* to Ca^2+^, of Δ*pmcC* to EDTA and of both to dithiothreitol. Regardless of more or less involvement in the Ca^2+^ homeostasis, all CA pumps were found participating in a variety of physiological and cellular processes, thereby exerting profound impact on the fungal adaptation to environment and host, as discussed below.

First, intracellular Ca^2+^ homeostasis is governed by a complicated system that comprises calcium pumps, channels and exchangers for calcium influx and efflux^21–24^. Characterized in this study, all CA pumps are distinctly classified to the PMCA, SERCA and SPCA groups and probably localized to different cellular compartments. Many previous reports have unveiled an increase of cellular sensitivity to Ca^2+^ in the absence of a CA gene, such as *eca1* ^10, 25^, yeast *pmc1* ^4, 26^ or filamentous fungal *pmcA*/*B* ^7, 9^, and *spf1* ^5, 6^. In this study, we found that most CA mutants were more sensitive to not only Ca^2+^ but also Fe^2+^, Cu^2+^, Zn^2+^ and the chelator EDTA. Exceptionally, both Δ*pmr1* and Δ*eca1* mutants showed a null response to the Ca^2+^ stress, and the same situation occurred in the Δ*pmcA* response to Fe^2+^, Cu^2+^ and Zn^2+^. Cellular sensitivity to Mn^2+^ increased in Δ*eca1* and Δ*spf1* but uniquely decreased in Δ*pmr1*. The responses of these CA mutants to the Ca^2+^ stress are consistent with the changes of Ca^2+^ levels in their cells. In addition, *B*. *bassiana* has three Pmc paralogues, contrasting to only a single Pmc1 acting as a vacuolar Ca^2+^ pump with Pmr1 in *S*. *cerevisiae*. Previously, a reduction of vacuolar Ca^2+^ pool in the absence of *pmc1* resulted in poor yeast growth under a Ca^2+^ stress to activate calcineurin^4^. In this study, the growth of three Δ*pmc* and Δ*pmr1* mutants was retarded by CsA at the optimum 25 °C or the high temperature 30 °C, suggesting a block of cytosolic Ca^2+^ transport into vacuoles when their calcineurin pathway was inactivated in the presence of CsA. The growth of Δ*eca1* and Δ*spf1* at 30 °C was also slowed down by CsA, hinting a possible block of the Ca^2+^ transport into the ER and Golgi apparatus.

Aside from differential responses to Ca^2+^ and other metal ions, the CA mutants were also differentially responsive to the ER stress. Cellular sensitivity to an ER stressor is relevant to calcium homeostasis. Tunicamycin is a nucleoside antibiotic that blocks *N*-linked glycosylation and the formation of *N*-glycosidic protein-carbohydrate linkages^27^ while dithiothreitol can also affect *N*-glycosylation by blocking protein folding in the ER^11^. In this study, all CA mutants were more sensitive to tunicamycin, and four of them, not including Δ*pmcA* and Δ*pmcC*, were more sensitive to dithiothreitol, coinciding well with the performance of *U*. *maydis* Δ*eca1* and *C*. *albicans* Δ*spf1* ^6, 11^. On the other hand, intracellular Ca^2+^ level increased in five CA mutants, not including Δ*pmr1*, under the stress of tunicamycin but only in three Δ*pmc* mutants under the stress of dithiothreitol. These results implicate that all CA pumps of *B*. *bassaiana* are involved in the *N*-glycosylation process of proteins and that most of them are functional in sustaining a relationship of calcium homeostasis with the ER stress despite some exceptions. Additionally, the fact that delayed culture acidification concurred with reduced levels of extracellular lactic and oxalic acids in three CA mutants provides a novel insight into prominent roles for Spf1, Pmr1 and PmcB in balancing ambient pH for the insect pathogen.

Moreover, all CA pumps play differential roles in the *in vitro* asexual cycle of *B*. *bassiana* because their deletion mutants showed moderate to severe defects in conidial germination, vegetative growth and/or aerial conidiation under normal culture conditions. The fungal growth defects on the rich medium and each of minimal media with different carbon/nitrogen sources were most severe in Δ*pmr1*, followed by Δ*spf1*, Δ*eca1* and Δ*pmcB* in order, but were inconspicuous in Δ*pmcA* and Δ*pmcC*. These observations are in accordance with the growth defects observed in the Δ*eca1* and Δ*spf1* mutants of some fungi^6, 11, 28^ but different from the normal growth of other fungal Δ*eca1* mutants^9, 10, 25^. Similarly, the growth defects were also observed in the previous deletion mutants of some PMCA genes (Δ*pmcA* and Δ*nca*-*2*) but not in the Δ*pmcB* and Δ*nca*-*3* mutants of *A*. *fumigatus* ^7^ and *N*. *crassa* ^9^ or in the Δ*pmc1* mutants of yeasts^4, 26^. The conidiation capacity of *B*. *bassiana* was most impaired in Δ*pmr1*, followed by Δ*spf1*, Δ*eca1* and Δ*pmcA*, but was less defective in Δ*pmcC* and Δ*pmcB*. Such phenotypic changes are similar to defective or weakened sporulation in *N*. *crassa* Δ*nca*-*2* and *S*. *cereverisiae* Δ*spf1* but are very different from unchanged sporulation in the Δ*nca-1*, Δ*nca-3* and Δ*pmc1* mutants of the two fungi^4, 9, 29^. In addition, aerial conidia produced by the fungal CA mutants required longer time for 50% germination, and most of the mutants, particularly Δ*spf1*, Δ*eca1* and Δ*pmcA*, lost conidial viability much more rapidly than their control strains during culture storage at 4 °C. Taken together, CA pumps are important for vegetative growth, aerial conidiation and conidial germination of *B*. *bassiana* but some of them, particularly the Pmc homologues, may functionally vary with fungal species.

Furthermore, all CA pumps play vital roles in sustaining antioxidant activity and cell wall integrity of *B*. *bassiana*. This is indicated by remarkable changes in the absence of each, including increased sensitivities to two oxidants and two cell wall perturbing agents, reduced activities of intracellular SODs and catalases, and altered cell wall components. Such changes have been rarely shown in previous CA studies. Total activities of SODs and catalases are crucial for the responses of *B*. *bassiana* to menadione and H_2_O_2_^30–32^ and were linearly correlated with antioxidant responses of all tested strains in this study. Fungal cell wall integrity is required for various cellular events, such as protein glycosylation, glycosyl-phoshatidylinositol (GPI) anchor biosynthesis, quality control of secretory proteins and delivery of cell wall components^33, 34^. These events are associated with the functions of all CA pumps because cell wall components, such as α-GlcNAc, β-GlcNAc and mannose residues, were altered due to the function loss of each CA pump. The impairment of cell wall integrity indicated by the altered cell wall components helps to understand the increased sensitivities of all CA mutants to cell wall perturbation by Congo red and SDS. Previously, only the Δ*spf1* mutant of *S*. *cerevisiae* has been shown an increased sensitivity to caffeine, another cell wall perturbing agent^29^.

Finally, the biological control potential of *B*. *bassiana* against insect pests and outdoor stresses is sustained by all CA pumps. The fungal virulence through normal cuticle infection and conidial UV-B resistance were attenuated in the absence of each CA pump. Conidial thermotolerance decreased in Δ*pmr1*, Δ*spf1* and Δ*pmcB*, increased in Δ*eca1* and Δ*pmcC* and was unchanged in Δ*pmcA*. Our results are consistent with attenuated virulence attributed to the respective deletions of *eca1*, *pmcA* and *spf1* in other fungal pathogens^6, 7, 10^. The attenuated pest control potential of the insect pathogen is apparently attributable to multi-phenotypic changes attributed to inactivated CA pumps, which affected not only intracellular Ca^2+^ homeostasis and cellular responses to several metal ions but also life cycle, antioxidant activity, cell wall integrity and extracellular pH balance in *B*. *bassiana*. Therefore, our results unveil a significance of each CA pump for the life of a filamentous fungal pathogen.

## Methods

### Microbial strains and culture conditions

The WT strain and its mutants were cultivated in SDAY (4% glucose, 1% peptone and 1.5% agar plus 1% yeast extract) for normal growth and conidiation at 25 °C in a light/dark cycle of 12:12 h and in 1/4 SDAY or CZA (3% sucrose, 0.3% NaNO_3_, 0.1% K_2_HPO_4_, 0.05% KCl, 0.05% MgSO_4_ and 0.001% FeSO_4_ plus 1.5% agar) for phenotypic experiments. *Escherichia coli* DH5α from Invitrogen (Shanghai, China) was grown in Luria-Bertani medium at 37 °C for plasmid propagation.

### Bioinformatic analysis of CA homologues in *B*. *bassiana*

All CA sequences of *A*. *fumigatus*, *C*. *albicans* and *S*. *cerevisiae* in NCBI database were used as queries to search through the *B*. *bassiana* genome database under the NCBI accession NZ_ADAH00000000^15^. The domains of each located homologue were predicted via online blast analysis at http://blast.ncbi.nlm.nih.gov/blast.cgi, followed by sequence alignment for phylogenetic analysis in parallel with the counterparts of some other fungi using a neighbor-joining method in MEGA7 software at http://www.megasoftware.net.

### Generation of CA mutants

Each CA-coding gene was deleted from the WT and rescued using the same protocols for *pmr1* deletion and complementation as described previously^12^. Briefly, the 5′ and 3′ coding/flanking fragments of each CA gene were amplified from the WT via PCR with paired primers (Table S1) under the action of an La*Taq* DNA polymerase (Promega, Madison, MI, USA) and inserted into the respective sites of two pairs of restriction enzymes (Table S1) in p0380-bar. The resultant plasmid p0380-5′*x*-bar-3′*x* (*x* = *eca1*, *pmcA*, *pmcB*, *pmcC* or *spf1*) was transformed into the WT for targeted gene deletion through homologous recombination. Subsequently, the full-length coding sequence of each gene with flanking regions was cloned from the WT with paired primers and ligated into p0380-sur-gateway to replace the gateway fragment, yielding p0380-sur-*x*. The new plasmid was ectopically integrated into the deletion mutant of each CA gene. Putative mutant colonies were screened in terms of the *bar* resistance to phosphinothricin (200 μg/ml for deletion mutants) or the *sur* resistance to chlorimuron ethyl (10 μg/ml for complement mutants) in a selection medium. These mutants were sequentially identified via PCR with paired primers, Southern blot hybridization with amplified probes and restriction enzymes and qRT-PCR with paired primers (Table S1). For qRT-PCR analysis, the WT and mutant strains were cultivated for 3 days at 25 °C on cellophane-overlaid SDAY (CO-SDAY) spread with 100 μl of a 10^7^ conidia/ml suspension per plate for culture initiation. Total RNAs were extracted from the hyphal cultures under the action of an RNAiso^TM^ Plus Reagent (TaKaRa, Dalian, China) and reversely transcribed into cDNAs with a PrimeScript® RT Reagent Kit (TaKaRa). Three samples of each cDNA (10-fold dilution) were used as templates to quantify transcripts of all CA genes in each strain under the action of SYBR® Premix Ex Taq^TM^ (TaKaRa) via qRT-PCR. The fungal 18 S rRNA was used as an internal standard. The transcript level of each gene in each cDNA was assessed using the 2^−ΔΔCt^ method^35^. Relative transcript level of each CA gene was calculated as the ratio of its transcript in each deletion mutant over that in the WT.

All positive deletion mutants and control strains were used in the following experiments of three replicates. The Δ*pmr1* and Δ*pmr1::pmr1* mutants constructed previously^12^ were also included in some experiments which were not carried out in the previous study.

### Assessment of intracellular Ca^2+^ concentration

Relative levels of intracellular free Ca^2+^ concentrations were assessed using the method of Fura-2-AM, a highly sensitive dye for rapid assessment of calcium flux in cells^7^, with a slight modification. Briefly, aliquots of 1 ml 10^7^ conidia/ml suspension in CZB were incubated by shaking at 25 °C for 8 h. The cells collected from the cultures by centrifugation were washed repeatedly with sterile water and resuspended in an equal volume of fresh CZB alone (control) or supplemented with CaCl_2_ (0.5 M), EDTA (2 mM), the ER inhibitor dithiothreitol (10 mM) or tunicamycin (5 µg/ml) as a treatment, followed by 30 min shaking at 25 °C. The treated and untreated cells were collected as above, washed three times with PBS (pH 8.0) and resuspended into 1 ml of 10 mM Fura-2-AM (Invitrogen) in the buffer. After 30-min staining at 37 °C and rinsing repeatedly, Fura-2-AM fluorescence was quantified from the stained cells at the alternating excitation wavelengths of 340/380 nm with an emission wavelength fixed at 505 nm. Relative level of free Ca^2+^ concentration in the stained cells was calculated as the ratio of fluorescence intensities (FI) at the two excitation wavelengths, i.e., FI_340_/FI_380_. The assay of each strain included three independent cell samples.

### Assays for cellular responses to metal ions, ER inhibitors and calcineurin inhibitor

Aliquots of 1 μl 10^6^ conidia/ml suspension were spotted centrally onto the plates (9 cm diameter) of minimal CZA alone (control) or supplemented with each of the chemical agents: (1) CaCl_2_ (0.5 M), ZnCl_2_ (10 mM), MnCl_2_ (5 mM), CuCl_2_ (2 mM), FeCl_3_ (3 mM), and EDTA (2 mM), a chelator of metal ions expected to be similar to metal ion starvation; (2) dithiothreitol (10 mM) and tunicamycin (5 µg/ml); and (3) the calcineurin inhibitor CsA (25 ng/ml). All the plates were incubated for 8 days at 25 °C (and at 30 °C only for the CsA treatment). The mean diameter of each colony was estimated from two measurements taken perpendicular to each other across the colony center. Relative growth inhibition (%) of each strain by each chemical treatment versus the control was calculated as (*A*_c_–*A*_t_)/*A*_c_×100, where *A*_t_ and *A*_c_ are colony areas from the treatment and control respectively.

### Quantification of pH and organic acids in liquid cultures

Aliquots of 100 ml 10^4^ conidia/ml suspension in CZB (initial pH = 7.3) were incubated by shaking (120 rpm) at 25 °C for 7 days. Samples of 10 ml were taken daily from three cultures (replicates) of each strain and filtered through filter paper. The pH level in each supernatant was assessed using an electronic pH detector. When pH levels differed greatly among the tested strains on day 5, concentrations of several organic acids (µg/ml) in each supernatant were quantified via ion-exchange chromatography on a Dionex ICS-2000 (Dionex Corporation, Sunnuvale, CA, USA), as described previously^36^.

### Assessments of growth rate, conidiation capacity, and conidial viability

Each strain was grown on the plates (9 cm diameter) of rich SDAY, minimal CZA and 11 modified CZA media using the same spotting method. The modified media were prepared by deleting 3% sucrose (carbon starvation) or 0.3% NaNO_3_ (nitrogen starvation) from CZA, replacing 3% sucrose with 3% of glucose, galactose, lactose, frucose, trehalose, glycerol or acetate (NaAc) as sole carbon, and replacing 0.3% NaNO_3_ with 0.3% of NaNO_2_ or NH_4_Cl as sole nitrogen, respectively. After 8 days of incubation at 25 °C and 12:12 h, the mean diameter of each colony was estimated as an index of growth rate, as described previously.

To assess aerial conidiation capacity of each strain, three aliquots of 100 μl 10^7^ conidia/ml suspension were evenly spread onto SDAY plates (9 cm diameter), followed by 7 days of incubation at 25 °C and 12:12 h. From day 4 onwards, culture plugs (5 mm diameter) were taken daily from the plates. Conidia on each plug were released into 1 ml of 0.02% Tween 80 via thorough vortex vibration. The conidial concentration in the suspension was determined using a haemocytometer and converted to *N* conidia/cm^2^ plate culture. In addition, conidia from fresh 7-day-old cultures or from those stored at 4 °C for 15 and 30 days were incubated at 25 °C for 24 h on the plates of a germination medium (2% sucrose and 0.5% peptone plus 1.5% agar), which were spread with the aliquots of 100 conidial suspension. During incubation, germination percentage on each plate was assessed using three microscopic counts. Conidial viability was estimated as time length required for 50% germination (GT_50_) or final germination percentage.

### Assays for cellular responses to abiotic stresses

Hyphal mass discs (5 mm diameter) were taken from 3-day-old CO-SDAYcultures, which were initiated by spreading 100 μl conidial suspension per plate, were attached centrally to the plates (90 mm diameter) of 1/4 SDAY alone (control) or supplemented with the gradients of the oxidants menadione (2–8 mM) and H_2_O_2_ (20–80 mM) or with a sensitive concentration of the cell wall perturbing agents Congo red (1.5 mg/ml) and sodium dodecyl sulfate (SDS; 0.5 mg/ml), respectively. After 6 days of incubation at 25 °C, diameters of all colonies were measured as described previously. For each strain exposed to each cell wall stressor, relative growth inhibition was calculated as (*A*_c_ − *A*_t_)/(*A*_c_ − d) × 100, where *A*_t_ and *A*_c_ are colony areas as described previously, and d is a constant area for the hyphal discs used for colony initiation. For each strain exposed to multiple concentrations of each oxidant, an effective concentration (EC_50_) required to suppress 50% colony growth was estimated as an index of antioxidant response by modeling analysis.

Conidial sensitivities to oxidative and cell wall perturbing stresses were assayed by spreading 100 μl aliquots of conidial suspension onto the plates of GM alone (control) or supplemented with a sensitive concentration of menadione (0.2 mM), H_2_O_2_ (4 mM), Congo red (1 mg/ml) or SDS (0.4 mg/ml). After 24 h incubation at 25 °C, percent germination on each plate was assessed using three microscopic counts. Relative germination was calculated as the ratio of percent germination under each stress over that in the control. Conidial responses to 45 °C wet-heat stress of 15–120 min and UV-B irradiation (weighted wavelength of 312 nm) of 0.1–0.8 J/cm^2^ were assayed as described previously^12^, resulting in the LT_50_ and LD_50_ estimates for conidial thermotolerance and UV-B resistance, respectively.

### Bioassay for fungal virulence

All deletion mutants and control strains were bioassayed on the larvae of *G*. *mellonella* as a model insect host. Cohorts of 30–40 larvae (~300 mg *per capita*) from a vendor (Da Mai Chong Insectaries, Wuxi, Jiangsu, China) were immersed in 30 ml aliquots of 10^7^ conidia/ml suspension of each strain (treatment) for ~10 s, transferred onto a towel paper to remove excessive water and then maintained in Petri dishes (15 cm diameter) for up to 10 days at 25 °C. During the period, larval mortality in each dish was monitored every 24 h. Time-mortality trends from the three replicates of each bioassay were subjected to probit analysis, yielding LT_50_ (*N* days) estimates of each strain against the insect species.

### Assays for total activities of intracellular antioxidant enzymes

Protein samples were extracted from three aliquots (replicates) of 0.5 g fresh hyphal cultures of each strain grown on CO-SDAY for 3 days at 25 °C by suspending ground samples in 50 mM phosphate buffer (pH 7.4). After centrifugation at 16,000 × *g* for 20 min at 4 °C, protein concentration (mg/ml) in each supernatant was determined using a BCA Protein Assay Kit (KeyGen, Nanjing, China). Total activities of SODs and catalases in the supernatant were assessed using an SOD Activity Assay Kit (Sigma) and a Catalase Activity Assay Kit (Jiancheng Biotech, Nanjing, China), respectively. The principles and procedures for the enzyme assays and the methods for standardizing enzyme activity to the number of units per milligram of protein extract (U/mg) were described in our previous study^37^.

### Quantification of carbohydrate epitopes on conidial walls

Carbohydrate epitopes on the surfaces of aerial conidia from the SDAY cultures were probed with the Alexa fluor 488-labeled lectins ConA (specific to α-glucose and α-GlcNAc), GNL (specific to mannose residues) and WGA (specific to β-GlcNAc and sialic acids) from Molecular Probes-Invitrogen and Vector Laboratories following previous protocols^38, 39^. Briefly, conidia were fixed in 3% formaldehyde for 30 min, washed three times with PBS buffer (a mixture of 137 mM NaCl, 2.7 mM KCl, 8.1 mM K_2_HPO_4_ and 1.5 mM KH_2_PO_4_, pH 7.4), and resuspended in the PBS containing 0.02% Tween 80, followed by centrifugation for collection. The pre-treated conidia were labeled for 1 h in darkness with ConA (60 µg/ml), WGA (20 µg/ml) and GNL (20 µg/ml) in each lectin-binding buffer (the user’s guide). The labeled conidia were washed five times in the binding buffer to remove unbound lectin. Fluorescent intensity in 2 × 10^4^ labeled conidia was quantified on the flow cytometer FC500 MCL (Becman Coulter, Inc., Brea, CA, USA) with an argon laser at the excitation/emission wavelengths of 488/530 nm ( ±15 nm). Data were collected and sorted with CELL QUEST and FACS EXPRESS V3 software at http://www.denovosoftware.com. The lectin-binding assay of each strain included three independent samples.

### Statistical analysis

All phenotypic parameters from the repeated experiments were subjected to one-factor (strain) analysis of variance, followed by Tukey’s honestly significant difference (HSD) test for the means of each phenotype between each deletion mutant and its control strains.

## Electronic supplementary material

Figures S1 and S2 and Table S1
