# Supplementary material for: Differential Roles for Six P-Type Calcium ATPases in Sustaining Intracellular Ca2+ Homeostasis, Asexual Cycle and Environmental Fitness of Beauveria bassiana
Source: Sci Rep. 2017 May 3;7:1420. doi: 10.1038/s41598-017-01570-1 (PMC5431182; doi:10.1038/s41598-017-01570-1)
Supplement: Supplementary file 1 — Figures S1 and S2 and Table S1 [file 41598_2017_1570_MOESM1_ESM.pdf]

Scientific Reports

Supplementary Information

Differential Roles for Six P-Type Calcium ATPases in Sustaining Intracellular Ca^{2+} Homeostasis, Asexual Cycle and Environmental Fitness of *Beauveria bassiana*

Jie Wang[1,2], Xiao-Guan Zhu[1], Sheng-Hua Ying[1], and Ming-Guang Feng [1]*

[1] Institute of Microbiology, College of Life Sciences, Zhejiang University, Hangzhou, Zhejiang, 310058, China (*Corresponding at: mgfeng@zju.edu.cn)

[2] Key Laboratory of Tropical Marine Bio-resources and Ecology, RNAM Center for Marine Microbiology, Guangdong Key Laboratory of Marine Material Medical, South China Sea Institute of Oceanology, Chinese Academy of Sciences, Guangzhou, 510301, China

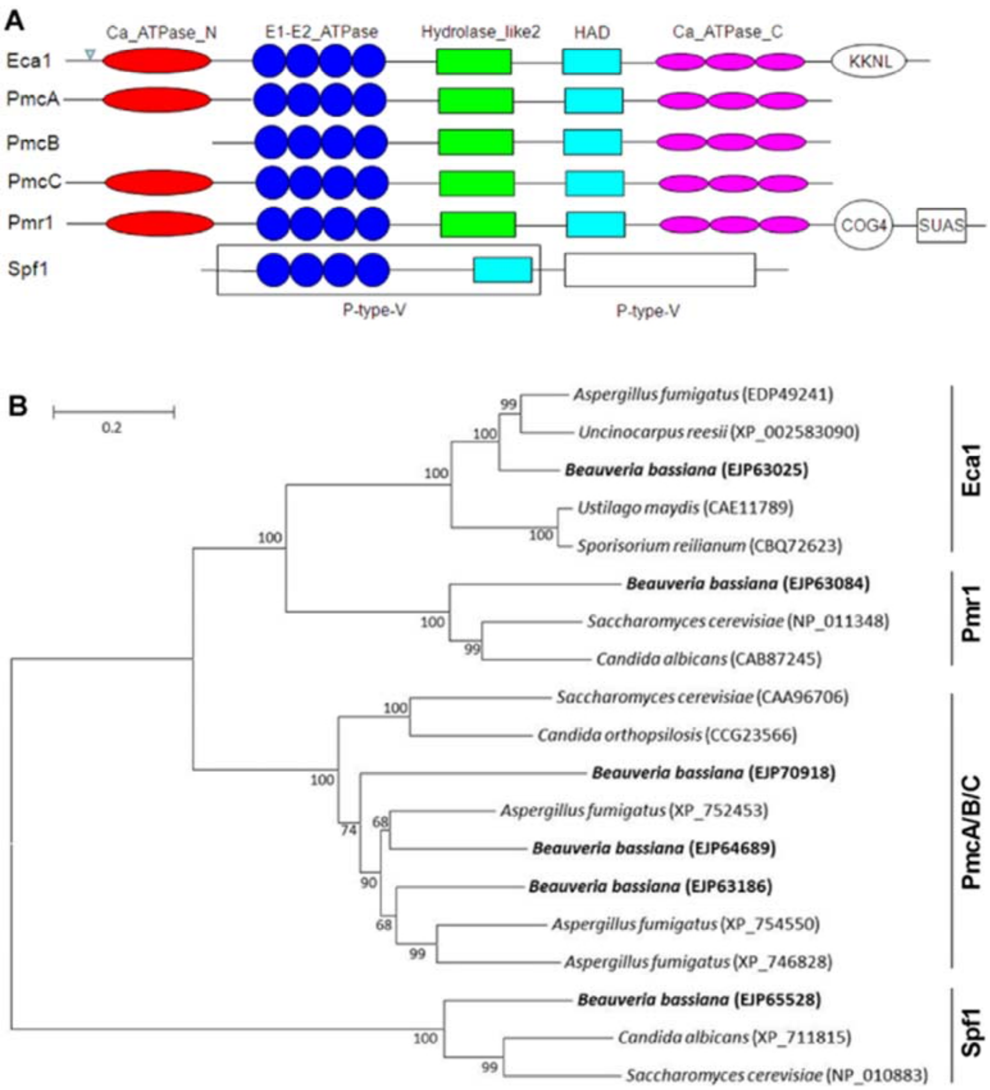

Figure S1 Structural and phylogenetic features of six P-type calcium ATPases found in the genome database of *B. bassiana*. (A) Conserved domains predicted from the calcium ATPases via sequence alignment and online blast analysis at http://blast.ncbi.nlm.nih.gov/blast.cgi. (B) Phylogenic tree constructed for the calcium ATPases in *B. bassiana* and other fungi (NCBI accession codes given in parentheses following the fungal names) using a neighbor-joining method in MEGA7 software at http://www.megasoftware.net. Scale bar: branch length proportional to genetic distance.

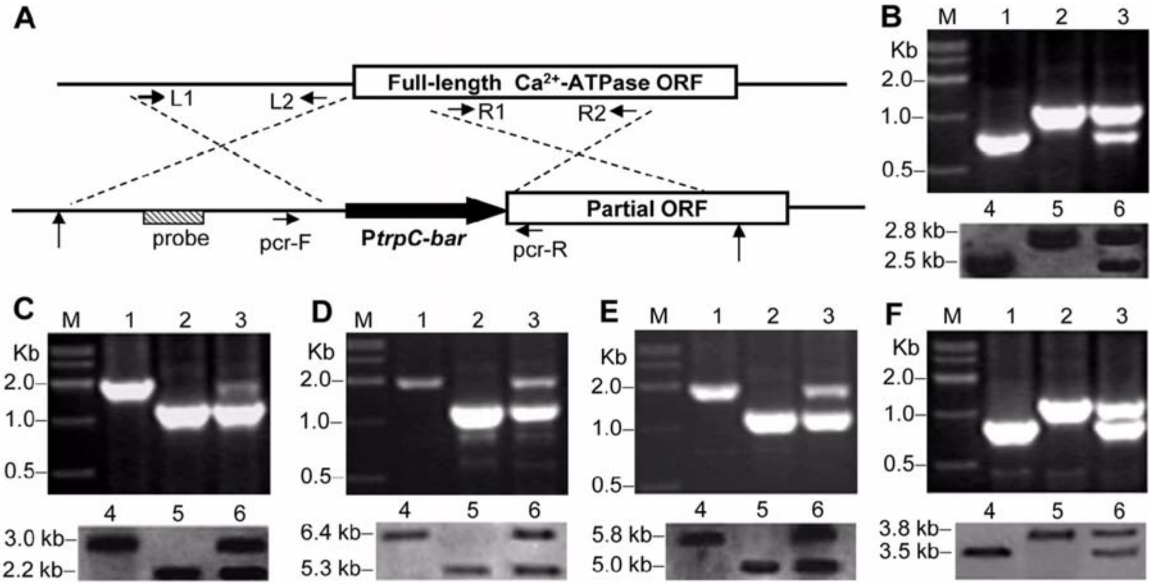

Figure S2 Constructing and identifying the deletion/complement mutants of calcium ATPase genes in *B. bassiana*. (A) Schematic diagram for the deletion strategy of each target gene. The upward arrows indicate the sites of restriction enzymes used for Southern blotting of *eca1* (*Eco*RI/*Eco*RI), *pmcA* (*Eco*RV/*Eco*RV), *PmcB* (*Eco*RV/*Eco*RV), *pmcC* (*Eco*RI/*Eco*RI) and *spf1* (Nde*I*/*Spe*I). (B–F) The mutants of *eca1*, *pmcA*, *pmcB*, *pmcC* and *spf1* identified via PCR (*lanes 1–3*) and Southern blot hybridization (*lanes 4–6*) respectively. *Lanes 1* and *4*: wild-type strain. *Lanes 2* and *5*: deletion mutant of each gene. *Lanes 3* and *6*: complemented mutant of each gene.

Table S1 Paired primers designed for the deletion, complementation and detection of calcium ATPase genes in *B. bassiana*.

Primer	Paired sequences (5′–3′)*	Purpose
Eca15’-F/R	AAAAAGGATCCTTCGGTCAGGTTTTGGTGGAT / AAAAAAAGCTTTTGGGGGATGCGTCTTGTGT	Cloning *eca1* 5′-end (1551 bp)
Eca13’-F/R	AAAAAACTAGTTATCATCTCCGTCTCCGTCG / AAAAAGTTAACCACGGGAGAACAAAGAGGC	Cloning *eca1* 3′-end (1565 bp)
PmcA5’-F/R	AAAAAGAATTCCTGGAAGACTGGGATAGCCT / AAAAAGGATCCGATGACAACGATGACGCCTC	Cloning *pmcA* 5′-end (1927 bp)
PmcA3’-F/R	AAAAAAGATCTGACTAAAAAGTGGATGGCTG / AAAAAGTTAACAGTGTGTTGAAGAACCCCAG	Cloning *pmcA* 3′-end (2103 bp)
PmcB5’-F/R	AAAAAGAATTCTGCCTTGTTCAGAGTTGGTG / AAAAAGGATCCAGGTTTGTTGGAAAGAGGCT	Cloning *pmcB* 5′-end (2065 bp)
PmcB3’-F/R	AAAAATCTAGACCGCCCAAAAAGGTCAGATG / AAAAAGTTAACGCCGAAGAAGACGATGAGGAC	Cloning *pmcB* 3′-end (1995 bp)
PmcC5’-F/R	AAAAAGAATTCTAGAGTCGTTGGTGGAAGTAT / AAAAAGGATCCTGTGGGTGTAGTCTTCTTCAT	Cloning *pmcC* 5′-end (1846 bp)
PmcC3’-F/R	AAAAAACTAGTAATCCGTCGCCATCAACTCG / AAAAAGTTAACCGTTTCTCATCCTCTCGTTTCTC	Cloning *pmcC* 3′-end (1913 bp)
Spf15’-F/R	AAAAAGAATTCCTTCCGAGAATGAGTCAGTCCC/AAAAAGGATCCTTGGGTCAAGAAAATAAAGGGC	Cloning *spf1* 5′-end (1913 bp)
Spf13’-F/R	AAAAAAGATCTACCACTATGGCACCAACACCTT/AAAAAGTTAACACGGCAGTCAGAGGATTGTCAC	Cloning *spf1* 3′-end (1696 bp)
Eca1fl-F/R	GGGGACCACTTTGTACAAGAAAGCTGGGTTTGGTGTTTGCGTCATCAGT / GGGGACAAGTTTG TACAAAAAAGCAGGCTCTTTGCCAATCTTGTCTCCT	Cloning full-length *eca1* (5888 bp) for complementation
PmcAfl-F/R	GGGGACCACTTTGTACAAGAAAGCTGGGTTCAGAACATCCGACAGAAGC / GGGGACAAGTTTG TACAAAAAAGCAGGCTAACAGAAACGCAAAAGAACC	Cloning full-length *PmcA* (6744 bp) for complementation
PmcBfl-F/R	GGGGACCACTTTGTACAAGAAAGCTGGGTCAGGCTTCGTCCTTTTATTC / GGGGACAAGTTTGT ACAAAAAAGCAGGCTATGGAGAAGACCTGGACAAG	Cloning full-length *PmcB* (7288 bp) for complementation
PmcCfl-F/R	GGGGACCACTTTGTACAAGAAAGCTGGGTAAATGAATCAGAGCGAGTGG / GGGGACAAGTTTG TACAAAAAAGCAGGCTCACATCCACAACACGAACTC	Cloning full-length *PmcC* (6552 bp) for complementation
Spf1fl-F/R	GGGGACCACTTTGTACAAGAAAGCTGGGTCCCTTCACAAACAGAGATGC / GGGGACAAGTTTG TACAAAAAAGCAGGCTCGTTGCTGTTGTTTTGACTC	Cloning full-length *Spf1* (6545 bp) for complementation
pEca1-F/R	TGTTCATCGTCAGCCAGCCT/CAATGACACGGCAATCGGC	PCR detecting *eca1*
sbEca1-F/R	TCGTCTCCTCATCTCGCAAG/AACGATTGAACCAAAGGCTG	Southern blotting *eca1*
qEca1-F/R	TTGATTCTGCTCGGTTCT/ATAAGAATGGTGAGGATAACAG	qRT-PCR detecting *eca1*
pPmcA-F/R	CCTTCTTTCTGTCTCTTGCCG/AATGATGGTGACGACGACGAT	PCR detecting *pmcA*
sbPmcA-F/R	AGCCAAAGTGCCGAGAATAG/GTGGCTTGAATGTCTGTGGT	Southern blotting *pmcA*
qPmcA-F/R	ATCGTAACATCTCCTCCT/CTGTCTCTGCTTTCAAAC	qRT-PCR detecting *pmcA*
pPmcB-F/R	CCTTCTTTCTGTCTCTTGCCG/AATGATGGTGACGACGACGAT	PCR detecting *pmcB*
sbPmcB-F/R	AGCCAAAGTGCCGAGAATAG/GTGGCTTGAATGTCTGTGGT	Southern blotting *pmcB*
qPmcB-F/R	ATCGTAACATCTCCTCCT/CTGTCTCTGCTTTCAAAC	qRT-PCR detecting *pmcB*
pPmcC-F/R	TATTTTTTCGGGGTTTTTCCTC/TGCCATCTTCCTCGCTCTCG	PCR detecting *pmcC*
sbPmcC-F/R	ATTTTCCAACCTGGCGAGAG/TTGACCAACGGGAACACCAC	Southern blotting *pmcC*
qPmcC-F/R	CACTTCGTCCTGATCCTG/TGACTTTGGGTTCAAGAG	qRT-PCR detecting *pmcC*
pSpf1-F/R	CCGAATCACCATCAGGTCCAT/AACGCAGAAGATTTGAAAGACG	PCR detecting *spf1*
sbSpf1-F/R	CGAGTGACATTATTTGCCAG/GCTGCTATCACCTACCTACC	Southern blotting *spf1*
qSpf1-F/R	CTGAATCCGCTTTCCTTT/ATGTGTTTCTCGTAGAGC	qRT-PCR detecting *spf1*
qPmr1-F/R	GTAGTGTCGCAGATGAAT / GTAAGTCCGTGAGTGAGG	qRT-PCR detecting *Pmr1*
q18S-F/R	TGGTTTCTAGGACCGCCGTAA / CCTTGGCAAATGCTTTCGC	qRT-PCR detecting 18S rRNA

* Underlined regions denote the sites of restriction enzyme for the deletion of each target gene (*Bam*HI/*Hin*dIII and *Spe*I/*Hpa*I for *eca1*, *Eco*RI/*Bam*HI and *Bgl*II/*Hpa*I for *pmcA*, *Eco*RI/*Bam*HI and *Xba*I/*Hpa*I for *pmcB*, *Eco*RI/*Bam*HI and *Spe*I/*Hpa*I for *pmcC*, and *Eco*RI/*Bam*HI and *Bgl*II/*Hpa*I for *spf1*) via homogenous recombination of 5′ and 3′ fragments separated by *bar* marker or the gateway exchange fragments for complementation of each target gene.
